# Once-Daily Subcutaneous Irisin Administration Mitigates Depression- and Anxiety-like Behavior in Young Mice

**DOI:** 10.3390/ijms24076715

**Published:** 2023-04-04

**Authors:** Patrizia Pignataro, Manuela Dicarlo, Clelia Suriano, Lorenzo Sanesi, Roberta Zerlotin, Giuseppina Storlino, Angela Oranger, Chiara Zecca, Maria Teresa Dell’Abate, Giorgio Mori, Maria Grano, Silvia Colucci, Graziana Colaianni

**Affiliations:** 1 Department of Translational Biomedicine and Neuroscience (DiBraiN), University of Bari Aldo Moro, Piazza Giulio Cesare 11, 70124 Bari, Italy; lorenzo.sanesi@uniba.it (L.S.); giuseppina.storlino@uniba.it (G.S.); silviaconcetta.colucci@uniba.it (S.C.); 2 Department of Precision and Regenerative Medicine and Ionian Area (DiMePRe-J), University of Bari Aldo Moro, Piazza Giulio Cesare 11, 70124 Bari, Italy; manuela.dicarlo@uniba.it (M.D.); clelia.suriano@uniba.it (C.S.); roberta.zerlotin@uniba.it (R.Z.); angela.oranger@uniba.it (A.O.); graziana.colaianni@uniba.it (G.C.); 3 Center for Neurodegenerative Diseases and the Aging Brain, Department of Clinical Research in Neurology, University of Bari at “Pia Fondazione Card G. Panico” Hospital, Via San Pio X, 4, 73039 Tricase, Italy; chiarazecca.cz@gmail.com (C.Z.); dellabatemariateresa@gmail.com (M.T.D.); 4 Department of Clinical and Experimental Medicine, University of Foggia, 71100 Foggia, Italy; giorgio.mori@unifg.it

**Keywords:** antidepressant, anxiolytic, irisin, subcutaneous injection, young mice, open field test, elevated plus maze, tail suspension test, forced swim test, Y-maze

## Abstract

Major depression is one of the most common psychiatric disorders worldwide, usually associated with anxiety. The multi-etiological nature of depression has increased the search for new antidepressant molecules, including irisin, for which, in a previous study, we tested its effect in young mice when administered intraperitoneally in a long-term intermittent manner. Here, we evaluated the effect of subcutaneous short-term irisin administration (100 µg/Kg/day/5 days) in male and female mice subjected to behavioral paradigms: Tail Suspension Test (TST), Forced Swim Test (FST), Elevated Plus Maze (EPM), and Y Maze (YM). Moreover, a qRT-PCR assay was performed to analyze the impact of irisin treatment on Pgc-1α/FNDC5 expression in the brain. A significant reduction in immobility time in TST and FST was observed in irisin-treated mice. Furthermore, irisin treatment significantly increased the number of entries and time spent in open arms, demonstrating its anxiolytic effect. Memory-enhancing effects were not reported in YM. Interestingly, no gender differences were observed in all behavioral tests. Overall, these results suggest that short-term subcutaneous irisin administration can exert an antidepressant and anxiolytic role, probably due to the activation of the Pgc-1α/FNDC5 system in the brain. Further investigation could lead to the identification of irisin as a new agent for the treatment of psychiatric disorders.

## 1. Introduction

The term “depression” is usually used to refer to a state of melancholy, emptiness, or agitation, accompanied by physical and cognitive symptoms that considerably impair the person’s functioning. Depression has become one of the most common mental illnesses worldwide, with a very high social cost [1,2,3,4]. It has been estimated that about one in six people develop at least one episode of severe depression in their lifetime [3,5]. According to the cognitive-behavioral approach, depressed people are characterized by a pessimistic view of themselves, the outside world, and the future [6]. However, even if many people may experience mood fluctuations, a single short-term depressive episode does not coincide with a diagnosis of severe/major depression, also known as Major Depressive Disorder (MDD). Indeed, MDD is defined as a disabling psychological condition, characterized by a persistent depression state that lasts for more than two weeks. This condition is often accompanied by other symptoms such as emotional distress, cognitive dysfunction (slowed speech, memory, attention, and decision-making difficulties), lack of interest and pleasure, loss of energy, sleep, appetite alterations, and health issues. In the most severe cases of MDD, the patients have recurrent death and suicidal thoughts or attempt suicide (up to 15% of untreated depression) [7,8,9].

In the last years, there was an increasing interest in the investigation of the mechanisms associated with the pathogenesis of MDD, but these studies are still in their infancy and the precise MDD etiology is still not fully elucidated. Several factors, including genetic, biochemical, environmental, female gender, physical inactivity, and psychological aspects, such as traumatic life experiences, have been involved in depression pathogenesis [9,10,11]. Additionally, stressful life events, such as the pandemic diffusion of COVID-19 virus, exacerbate the depression incidence as well as other psychiatric and/or mental disorders (such as anxiety, stress, and memory complaints) often associated with the MDD state [12,13]. Based on the current knowledge, chronic neuroinflammation and pain are additional important risk factors involved in depression [14,15,16].

The multifaceted pathophysiology of MDD has hampered the depression management and the success of therapeutic interventions [9]. Currently, many treatments, including pharmacological, psychological, and neurostimulatory approaches, have been proposed to achieve the symptom remission, but, in some cases, depressed patients are incapable of reaching and/or maintaining this condition. This situation is defined as “treatment resistant depression” (TRD) [14,17,18,19].

In the last decades, several bioactive molecules have been suggested to treat depressive disorders and other psychiatric and neurodegenerative conditions with similar pathogenic features and/or risk factors [20,21,22,23,24]. Recently, different behavioral studies based on rodents reported the potential antidepressant effect of irisin, a polypeptide of 112 amino acids released from the muscle cells into the bloodstream during physical exercise [25,26,27,28]. Employing behavioral paradigms for the screening of potential antidepressant compounds, i.e., Forced Swim Test (FST) and Tail Suspension Test (TST), the effect of irisin on mice and rats subjected to stress conditions was evaluated. Siteneski et al. demonstrated that an intracerebroventricular injection (i.c.v.) of irisin at a dose of 0.5 to 1 ng/mouse decreased the duration of the immobility time up to 6 h in the FST and the TST [26]. Similarly, a subcutaneous irisin injection (100 ng/mL) significantly reduced the immobility time in the FST and increased the sucrose preference in a dose-dependent manner in a Chronic Unpredictable Stress (CUS) rat model [27].

In line with these findings, in our previous work, the antidepressant effect of long-term intermittent intraperitoneal administration of irisin in young mice was shown. In particular, for the first time, it was demonstrated that the systemic administration of irisin (100 µg/kg/week for 1 month) significantly reduced the immobility time in the TST and the FST. Furthermore, the potential role of irisin in modulating anxiety behaviors was suggested by the increase in the time spent in the inner zone in the Open Field Test (OFT) [28].

Here, a new treatment strategy based on the short-term subcutaneous irisin administration for 5 days consecutively (100 µg/Kg/daily) was proposed to evaluate the antidepressant effect of the myokine in young healthy mice submitted to the TST and FST. Moreover, in order to investigate anxiety-like behavior and memory performances the Elevated Plus Maze (EPM) and Y-Maze (YM) were carried out, respectively. With the aim to evaluate the effect of irisin treatment on the brain FNDC5/irisin system, the gene expression of irisin precursor, Fndc5, and the transcriptional regulator that increases its expression (PGC-1α) were analyzed in the cerebral areas that are the most implicated in MDD, i.e., the hippocampus and the Prefrontal Cortex (PFC) [29,30].

## 2. Results

### 2.1. Short-Term Irisin Treatment Did Not Impact Mouse Locomotor Activities in the OFT

**Figure 1:**
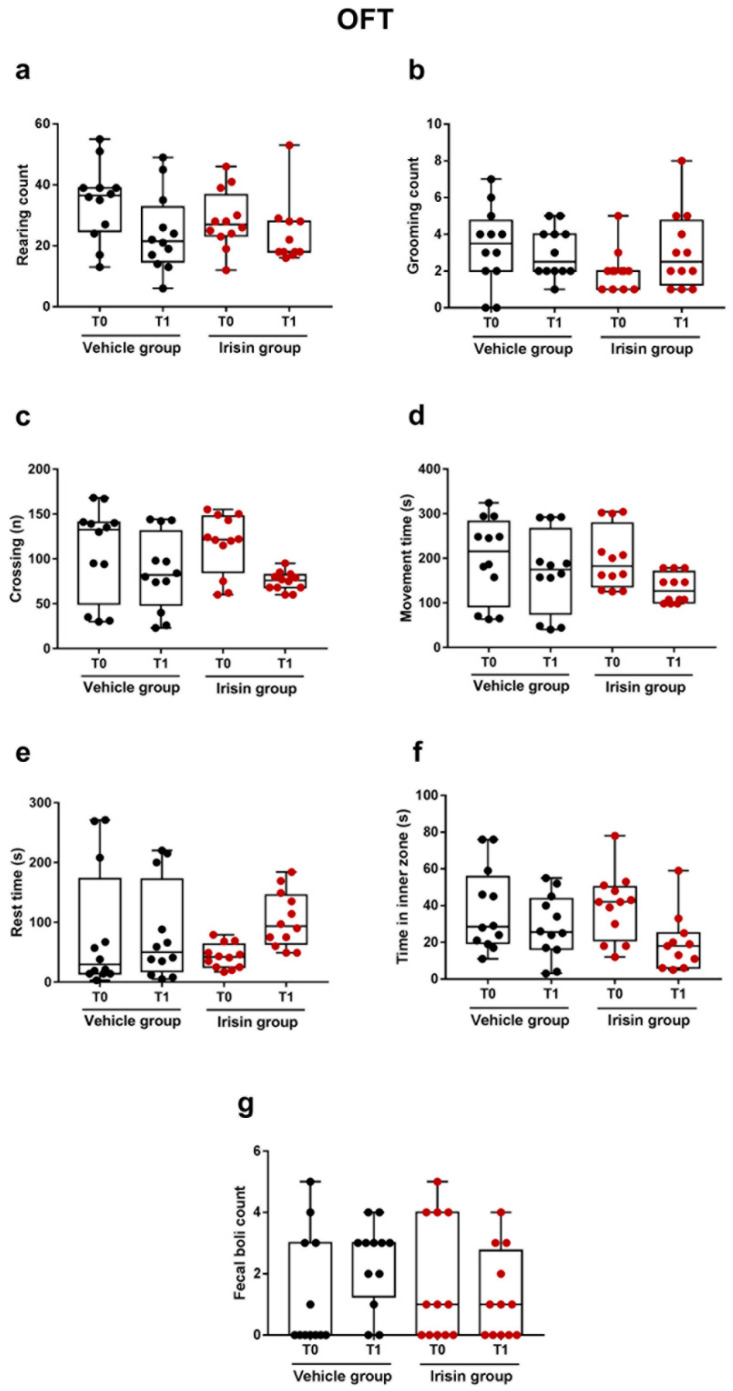
Effect of short-term systemic administration of irisin on the rearing count (**a**), grooming (**b**), crossing (**c**), movement time (**d**), rest time (**e**), time spent in inner zone (**f**), and fecal boli count (**g**) in the OFT before treatment (T0) and after two hours (T1) at day 5. Shapiro–Wilk test and unpaired Kruskal–Wallis test were used for statistical analysis. Data are presented as box-and-whisker with median and interquartile ranges, from max to min, with all data points shown. *n* = 12 mice per group.

The OFT was performed before treatment (T0, day 5) and 2 h after the fifth irisin injection (T1, day 5) to evaluate mouse locomotor abilities and behavior associated with emotionality. No significant differences were observed between the irisin-treated mice and the control group for all the behavioral parameters analyzed: rearing (*p* = 0.10), grooming (*p* = 0.19), crossing (*p* = 0.20), movement time (*p* = 0.11), rest time (*p* = 0.10), time spent in inner zone (*p* = 0.06), and fecal boli (*p* = 0.33) at both time points (Figure 1).

### 2.2. Short-Term Irisin Treatment Reduced Mouse Anxiety-like Behavior in the EPM

**Figure 2:**
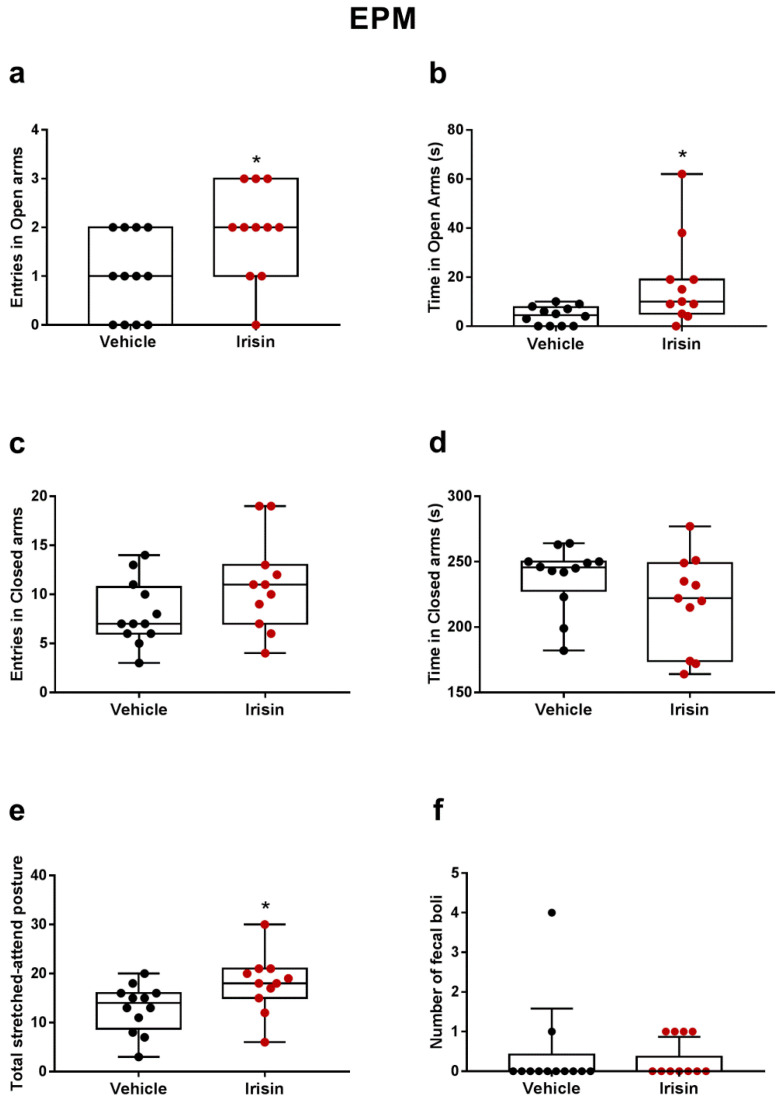
Effect of short-term systemic administration of irisin on the number of entries (**a**), time spent (**b**) in open arms, number of entries (**c**), time spent (**d**) in closed arms, total stretched-attend posture (**e**), and number of fecal boli (**f**) in the EPM. Shapiro–Wilk test and unpaired Mann–Whitney test were used for statistical analysis. Data are presented as box-and-whisker with median and interquartile ranges, from max to min, with all data points shown. *n* = 12 vehicle-treated mice and *n* = 11 irisin-treated mice. * *p* < 0.05.

The effect of the irisin treatment in the EPM is illustrated in Figure 2. A significant increase in both the number of entries (1.91-fold change, * *p* < 0.05) and time spent in open arms (3.98-fold change, * *p* < 0.05) was observed in the irisin-treated group compared with the control (Figure 2a,b). Conversely, no differences were noted between the two groups concerning the number of entries (*p* = 0.1003, Figure 2c) and time spent in closed arms (*p* = 0.1376, Figure 2d). A statistically significant difference in total stretched-attend postures was observed in the irisin-treated animals (1.39-fold change, * *p* < 0.05) (Figure 2e). The fecal boli count showed similar results in both experimental groups (*p* = 0.55) (Figure 2f).

### 2.3. Short-Term Irisin Treatment Decreased Mouse Depressive-like Behavior in the TST and FST

**Figure 3:**
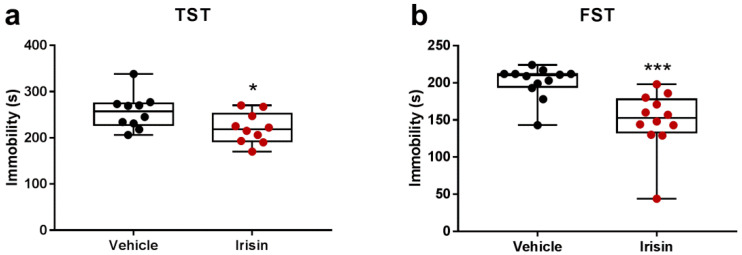
Effect of short-term systemic administration of irisin on the immobility time in the TST (**a**) and in the FST (**b**). Shapiro–Wilk test was followed by unpaired two-tailed Student’s *t*-test for the TST and unpaired Mann–Whitney test for the FST. Data are presented as box-and-whisker with median and interquartile ranges, from max to min, with all data points shown. *n* = 10 vehicle-treated mice and *n* = 10 irisin-treated mice for TST. *n* = 12 vehicle-treated mice and *n* = 12 irisin-treated mice for FST. * *p* < 0.05; *** *p* < 0.0001.

To examine the behavioral reactions of mice in stressful situations, the TST and FST were carried out. Results showed that the duration of immobility was considerably reduced in irisin-treated animals in both TST (−13.90%, * *p* < 0.05) and FST (−25.81%, *** *p* < 0.0001) (Figure 3a,b).

Additionally, we performed a comparison of the percentage change of the immobility time in the TST and the FST between the short-term and long-term irisin administration [28], summarized in Table 1.

### 2.4. Short-Term Irisin Treatment Produced Similar Effects on Mouse Spatial Working Memory in the YM

**Figure 4:**
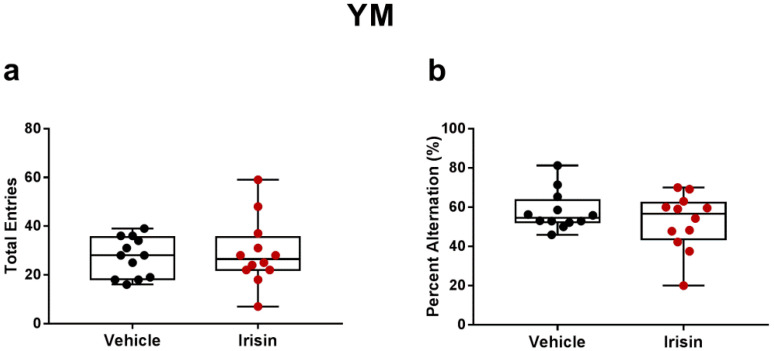
Effect of short-term systemic administration of irisin on the total entries (**a**) and in the percent alternation (**b**) in the YM. Shapiro–Wilk test was followed by unpaired two-tailed Student’s *t*-test. Data are presented as box-and-whisker with median and interquartile ranges, from max to min, with all data points shown. *n* = 12 vehicle-treated mice and *n* = 12 irisin-treated mice.

Spatial short-term memory was assessed to evaluate spontaneous exploratory behavior in the three arms of the Y-Maze. As shown in Figure 4, similar results between the vehicle and irisin groups were obtained from the analysis of the number of arm entries (*p* = 0.7075) and percent alternation (*p* = 0.2978) (Figure 4a,b).

### 2.5. Irisin Treatment Influenced the Behavioral Parameters

In order to evaluate the effect of irisin administration and sex on the behavioral parameters of the TST, FST, EPM, and YM, the two-way ANOVA analysis was performed. The effect of irisin treatment was highly significant for the immobility time in the TST (*p* = 0.042) and the FST (*p* = 0.002), the entries and time spent in the open arms (*p* = 0.043 and *p* = 0.041, respectively), and the total stretched-attend posture (*p* = 0.045) in the EPM. A main effect of sex was observed for the total stretched-attend posture in the EPM (*p* = 0.019) and the total entries in the YM (*p* = 0.001). There was a main effect of the interaction between treatment and sex for the entries in closed arms in the EPM (*p* = 0.039) and the percentage alternation in the YM (*p* = 0.022). All the results of this analysis are summarized in Table 2.

### 2.6. Irisin Short-Term Administration Increased Serum Irisin Levels

**Figure 5:**
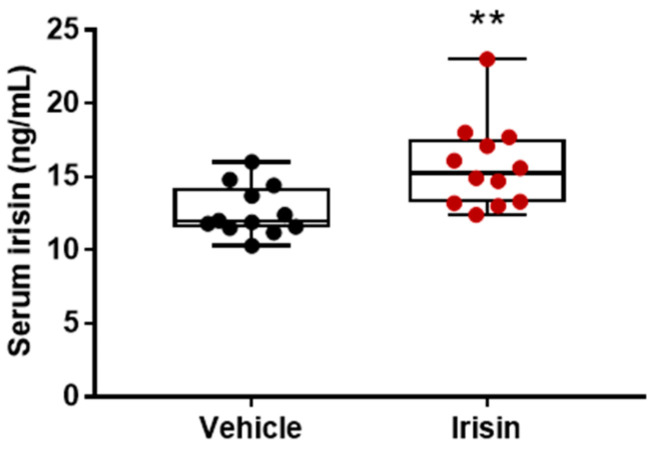
Effect of short-term systemic administration of irisin on serum irisin. Shapiro–Wilk test and unpaired two-tailed Student’s *t*-test were used for statistical analysis. Data are presented as box-and-whisker with median and interquartile ranges, from max to min, with all data points shown. *n* = 12 mice per group. ** *p* = 0.0045.

Subcutaneous administration of irisin for 5 days (100 µg/Kg/daily) significantly increased the serum irisin concentration. Irisin levels were 1.25-fold higher in irisin-treated mice compared with the control mice injected with vehicle (** *p* = 0.0045) (Figure 5).

### 2.7. Short-Term Systemic Administration of Irisin Enhanced the Gene Expression of Pgc-1α and Fndc5 in Both the Hippocampus and the PFC

**Figure 6:**
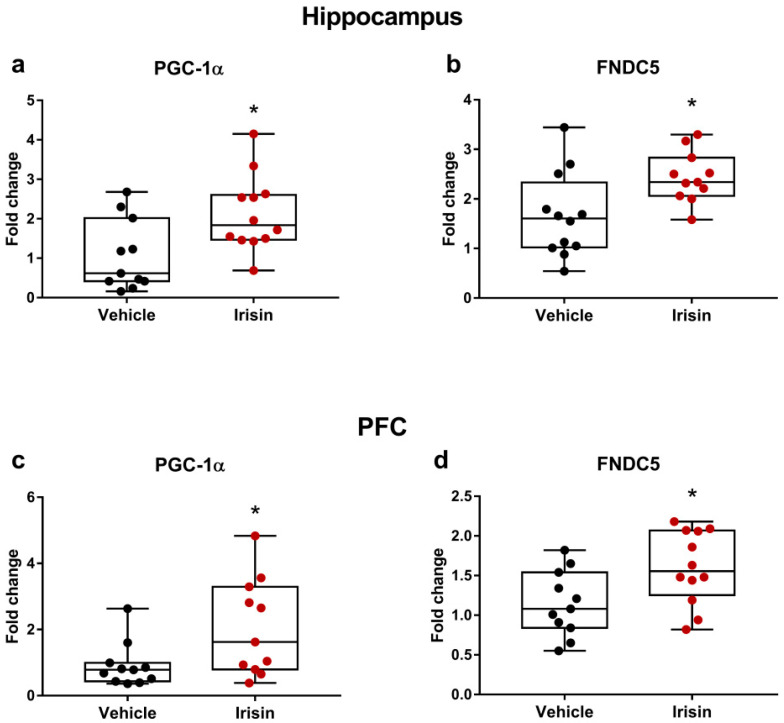
Effect of short-term systemic administration of irisin on FNDC5/irisin system in the hippocampus and the PFC. Gene expression of Pgc-1α (**a**,**c**) and FNDC5 (**b**,**d**) was assessed by qRT-PCR. Shapiro–Wilk test was followed by Student’s t test for all data except for Pgc-1α in the PFC. Data are presented as box-and-whisker with median and interquartile ranges, from max to min, with all data points shown. * *p* < 0.05.

To evaluate the effect of short-term irisin treatment on its precursor in the mouse hippocampus and the PFC, the gene expression of Fndc5 and Pgc-1α was analyzed by quantitative real-time PCR (qRT-PCR) assays. Irisin administration significantly increased the expression of Pgc-1α in both the hippocampus and the PFC (*p* < 0.05) (Figure 6a,c). The gene expression analysis of Fndc5 showed that daily irisin injections for 5 days induced a significant increase in both the cerebral regions (*p* < 0.05) (Figure 6b,d).

## 3. Discussion

In the present study, the potential antidepressant effect of irisin injection for 5 days consecutively (100 µg/kg/daily) was investigated in healthy young male and female mice, evaluating the behavioral responses by the TST and FST. These are the most used tests to examine depressive-like behavior and to screen antidepressant drugs [31,32,33]. Both assays assume that when the animal is subjected to a situation of extreme stress, which is one of the main factors in the propensity to severe depression, it will firstly try to escape but finally it will resign, developing an inertia attitude [34,35,36]. In the TST and the FST, the time spent in an immobility position was evaluated, an indicator of desperate behavior, i.e., a condition in which the animal shows a complete absence of movement (such as climbing, swimming, escaping, or scratching) [31,32,35,36]. The results of this investigation showed that the immobility time was significantly reduced in the irisin-treated mice compared with the control group, independently on the type and duration of the irisin treatment. Furthermore, in the FST, the irisin-treated mice displayed a pronounced swimming activity and, interestingly, the reduction of the immobility time was greater in this short-term protocol with respect to the long one (25% vs. 18%) [28]. Chatterjee and co-workers highlighted that the TST and FST induce the immobility in different manners and reported that the FST was a better model than TST to evaluate negative symptoms, such as depression, in psychotic mice [37]. In line with this, other studies evidenced sensitivity and performance differences in these paradigms [38,39,40]. To further validate our results in the TST and FST, as well as all the other paradigms carried out in the current study, we performed these tests two hours after irisin/vehicle administrations and considered the OFT results. Indeed, this assay was preliminary conducted at T0 before treatments in order to evaluate the locomotor activities of the mice [41,42], thus excluding possible excitatory effects of irisin treatment. The OFT showed that there were no differences in the exploratory locomotor activities such as rearing, crossing, and movement and rest time and in self-cleaning behaviors such as grooming. In addition, similar results were found in the time spent in the inner zone and in fecal boli excretion between the two experimental groups, suggesting a similar degree of emotionality [26,43].

Moreover, in the previous work, the results of the long-term irisin administration already suggested a possible anxiolytic effect of irisin as shown by the downward trend in the time spent in the inner zone of the OFT arena [28]. Therefore, considering that depression may be exacerbated and/or caused by anxiety disorders [44], in this study, the impact of once-daily short-term systemic irisin administration on anxiety behavior was also examined by the EPM. This assay is one of the most widely employed paradigms to test possible anxiolytic compounds in rodents and to measure the mouse propensity to explore open and closed spaces [45,46]. Our results evidenced that irisin treatment significantly increased the number of entries and the time spent in the open arms, suggesting that the irisin-treated mice are more prone to visit open spaces than the controls. In this test, concerning the number of entries and the time spent in closed arms, no differences were noted between the two groups. According to Albrechet-Souza and colleagues, these observations indicated that, even if the mice stayed longer in open arms, they also equally continued to explore closed places [47]. In addition, a statistically significant difference in total stretched-attend postures was noted in the irisin-treated mice. As indicated in the literature, this parameter implies an environmental risk assessment behavior occurring in a dangerous situation as an approach–avoidance conflict [48,49,50]. Moreover, the data of the stretched-attend posture scores suggested a reduction of internal conflict of avoidance of dangerous space in the irisin-treated mice, also demonstrated by increased entries count and the time spent in open arms.

MDD is often associated with memory impairment [24,51]. This condition represents a risk factor for the development of neurodegenerative diseases, including Alzheimer’s Disease [52,53]. Thus, several recent studies were carried out on animal models to identify the common biological mechanisms underlying both depression and neurodegenerative diseases [24,54]. Here, the YM was performed in order to evaluate the short-term memory abilities in mice, measuring the exploratory activity by the spontaneous alternation based on the consecutive entries in three arms. A mouse without memory deficits will alternate entries, correctly remembering the previously visited arm [55]. The results of YM evidenced that the working memory of short-term irisin treated mice did not change compared with the control group. This finding could be due to the young age of the mice. Indeed, in line with these results, Esquivel et al. reported a significant difference in the percentage of incorrect alternations, indicative of memory defects only in aged mice [56].

Epidemiologic studies evidenced that the incidence of MDD in women is higher compared with men; in fact, it has been estimated that the risk of experiencing depressive disorders for women is twice that of men at the same age [57]. Moreover, the incidence of depressive states markedly increases during the reproductive years (from 25 to 44 years), when women face different transitional events inducing neuroendocrinological variations that influence the risk for depressive episodes, i.e., menstrual cycle, pregnancy, and perimenopausal period [58]. Gender differences also play a pivotal role in the comorbidities associated with depression, such as anxiety, that affect women at a higher rate than men [59]. As gender could determine different responses to depression treatment, in this study, both female and male mice were equally treated with irisin or vehicle and submitted to behavioral testing. Results showed that the behavioral parameters were not influenced by sex, with some marginal exceptions (i.e., the total stretched-attend posture in the EPM and the total entries in the YM). The finding that the effect of irisin treatment was highly significant for most of the behavioral parameters without gender differences is encouraging in view of its future application in humans.

In this study, the subcutaneous injection replaced the most common route of administration in mice, i.e., intraperitoneal. This kind of treatment, more feasible for future application in humans, was proved to be effective in increasing irisin circulating levels. Overall, in line with the findings obtained after long-term intermittent irisin administration for 1 month [28], the results of the current study showed that the myokine irisin injected subcutaneously according to a short-time schedule displayed an obvious antidepressant as well as anxiolytic effect in young healthy mice with no gender differences. However, irisin administration did not impact working memory, possibly due to the young age of the mice. Based on previous studies reporting that irisin can cross the blood–brain barrier increasing the expression of Pgc-1α and Fndc5 [60,61], the results shown herein confirmed that peripheral irisin significantly enhanced the expression of these genes in both the hippocampus and the PFC. Thus, it could be hypothesized that the activation of the FNDC5/irisin pathway in the brain regions mainly involved in mood disorders could contribute to the antidepressant and anxiolytic-like effects of irisin. However, further investigations will be necessary to better clarify the molecular mechanisms underlying the positive effect of irisin on mental disorders.

## 4. Limitations

In this study, the effects of short-term irisin administration were evaluated in young healthy mice subjected to stressful conditions. However, future studies will be performed on specific rodent models of depression, such as mice treated with LPS, socially isolated, etc., even if none of them fully recapitulate the complex pathogenesis of depression in humans [54].

## 5. Materials and Methods

### 5.1. Ethical Statement

Mice were maintained under standard conditions in a 12/12 h light/dark cycle, with access to water and regular diet ad libitum (Harlan Teklad 2019, SDS, England). The approval of this animal interventional study was obtained by the Italian Health Authority (aut. n° 12/2022-PR, obtained on 12 January 2022). The care and husbandry of the animals was performed in accordance with European Directives no. 2010/63 and with the Italian Regulatory system (D.L. vo n. 26, 4 March 2014). All parts of this study concerning animal care were approved by the animal welfare agency (OPBA) of the University of Bari.

### 5.2. Animals

**Figure 7:**
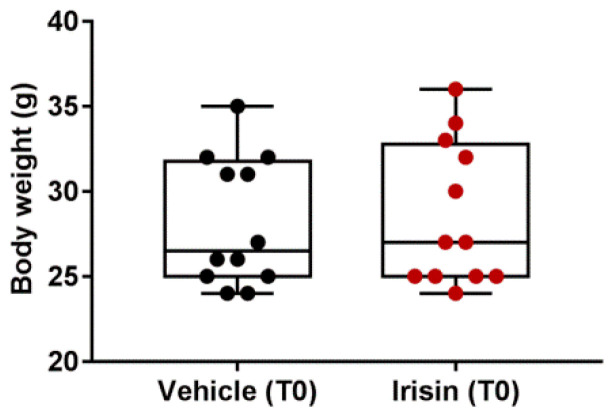
Body weight comparison between vehicle and irisin group before treatment (T0). Shapiro–Wilk test and unpaired two-tailed Student’s *t*-test were used for statistical analysis. Data are presented as box-and-whisker with median and interquartile ranges, from max to min, with all data points shown. *n* = 12 mice per group.

Twenty-four male (*n* = 10) and female (*n* = 14) black Swiss mice (C57BL/6), aged 22–25 weeks and weighing 23–34 g, were utilized in this study. They were housed in a room in typical rodent polypropylene cages with unlimited access to food and water. The environment was kept at a controlled noise, temperature (21 ± 1 °C), humidity (50 ± 20%), and lighting (12/12 h light/dark cycle, starting 6 a.m.). Animals were randomly divided into six home cages (4 per group) two weeks before the experiment starts. To stratify female and male mice into experimental groups, the body weight was recorded before starting the experiment (Figure 7).

### 5.3. Irisin Treatment

Mice received a total of 5 consecutive subcutaneous injections of recombinant irisin (r-irisin) (100 µg/kg/once-daily) (*n* = 12) or vehicle (physiological saline sterilized by 0.22 μ filtration) (*n* = 12). Injections were delivered at the same time each day. r-irisin was provided by Adipogen International (San Diego, CA, USA). The concentration of irisin was chosen based on previous studies by our research team [28,62].

### 5.4. Behavioral Tests

The Open Field Test (OFT), the Elevated Plus Maze (EPM), the Tail Suspension Test (TST), the Forced Swim Test (FST), and the Y-Maze (YM) were carried out in a blinded manner by a trained investigator during the light phase (11:00 a.m. and 16:00 p.m.). The groups of mice were brought into the testing room 30 min before each behavioral experiment to give them time to adapt to the new environment. The trials were conducted on independent groups of mice two hours after the last vehicle or irisin injection. To neutralize odor cues and to disinfect the equipment, the mazes were cleaned with a 70% ethanol solution after each trial. During all test sessions, the mice were isolated acoustically and visually to avoid interferences.

**Figure 8:**
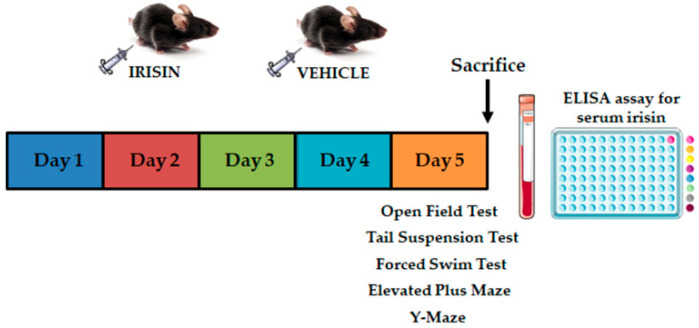
Experimental timetable. Time schedule of experimental design showed the behavioral tests performed after once-daily short-term protocol (5-day treatment), two hours after the fifth irisin or vehicle injection. ELISA assay for serum irisin was executed after mouse sacrifice.

Figure 8 showed the experimental timetable. Briefly, mice groups were submitted to the OFT, the EPM, the TST, the FST, and the YM after the last administration (day 5).

#### 5.4.1. OFT

The OFT was used to evaluate mouse locomotor activity and behaviors related to stress or anxiety according to the protocol described previously [41,42]. The device was an empty acrylic square arena (40 × 40 × 40 cm) with high opaque walls that prevented mice from jumping out. The animal was positioned in the middle of the arena, and during a 10-min observation, the number of rearings (unsupported and supported vertical exploration), groomings (general maintenance activity in which the animal cleans itself), square crossings, movement time, rest time, time spent in the inner zone, and the fecal boli total were evaluated.

#### 5.4.2. EPM

To measure exploratory and anxiety-related behaviors, the EPM was performed according to the method described by Lister [45].

A gray acrylic cross-shaped apparatus made up by two open arms with a 2 mm high border and two closed arms (both 35 × 6 cm) with walls 15 cm high raised 60 cm above the floor was used. In the test, the animal was placed in the maze center to allow its exploration for a 5-min session. The following parameters were recorded: the total number and the time spent in open/closed arms entries, the stretched-attend posture, and the fecal boli count.

#### 5.4.3. TST

In this paradigm, a predictive test of depressive behavior, mice were individually suspended by the tail approximately 1 cm from the tip for a short period of time (6 min) at 25 cm above the floor. The total duration of immobility, i.e., the condition in which the animal showed passivity without any movement, was recorded according to the protocol described previously by Steru L et al. [31].

#### 5.4.4. FST

The FST was carried out to examine the animal behavioral responses when subjected to forced swimming. Following the procedure used by Porsolt and coworkers [32,33], mice were individually placed in a transparent acrylic cylinder (diameter 13 cm, height 24.5 cm) filled with tap water (18 cm) at 26 °C. In this paradigm, after the initial 2 min of training, the total amount of time spent in a floating position without climbing or swimming with the nose above the water surface was measured during the last 4 min session.

#### 5.4.5. YM

The YM was performed to assess the spatial short-term memory by spontaneous alternation behavior according to a previous protocol [55]. The equipment consisted of a semi-transparent translucent acrylic y-shaped maze with three arms (35 cm long, 6 cm wide, and 15 cm high). Mice were independently located into the apparatus at the end of one arm and left to explore it for 8 min. The spontaneous alternation behavior, which is defined as the entering in all the three arms consecutively, was evaluated by recording the number of total entries. The percentage of alternation was calculated using the following formula: (number of alternations/total number of arm entries − 2) × 100.

### 5.5. Enzyme-Linked Immunosorbent Assay (ELISA) for Serum Irisin Measurement

Mouse blood samples were collected soon after the sacrifice of the animals and were centrifuged at 1000× *g* for 20 min at room temperature to collect serum. Circulating irisin was measured using a validated competitive ELISA kit (Cat. No. EK-067–29, Phoenix Pharmaceuticals, Burlingame, CA, USA), according to the manufacturer’s instructions. The assay sensitivity was 1.29 ng/mL, with a measurement range of 0.1–1000 ng/mL. Inter- and intra-assay variations were <15% and <10%, respectively. Serum samples, standard dilutions, and positive controls were run in duplicate and their absorbance was read at 450 nm on a plate reader (Eon, BioTek, Winooski, VT, USA). Results were reported in ng/mL.

### 5.6. Gene Expression Analysis by qRT-PCR Assays

After the behavioral tests, mice were sacrificed and brain dissections were performed. The hippocampal and the PFC tissues were carefully harvested and stored at −80 °C until further analysis. Total RNA extraction was carried out by Trizol (Invitrogen, Carlsbad, CA, USA), according to standard protocols. Complementary DNA (cDNA) was synthesized using 1 μg of RNA in 20 μL of reaction mixture, i.e., iScript Reverse Transcription Supermix (Bio-Rad Laboratories, Hercules, CA, USA) in a thermal cycler (My cycler; Bio-Rad Laboratories, Hercules, CA, USA). mRNA expression was measured by qRT-PCR using a CFX96 Real-Time System (Bio-Rad Laboratories, Hercules, CA, USA) with 10 μL reaction volume consisting of cDNA transcripts, primer pairs, and SsoFast EvaGreen Supermix (Bio-Rad Laboratories, Hercules, CA, USA). All primers were designed by Primer Blast (https://www.ncbi.nlm.nih.gov/tools/primer-blast/) accessed on 10 January 2023. Glyceraldehyde-3-phosphate dehydrogenase (GAPDH) was used as the reference gene. The oligonucleotide sequences for the target and reference genes are indicated in Table 3 The specificity of the qRT-PCR assays was detected by the melt curve analysis. All samples were assayed in triplicate and quantifications were normalized to a control gene in each reaction.

### 5.7. Data Analyses

The GraphPad Prism statistical software (version 7.0) was used to conduct the statistical analyses. To determine normal distribution of data sets, the Shapiro–Wilk normality test was used. Statistical significance of differences between vehicle- or r-irisin-treated mice was evaluated using the Student’s t or Mann–Whitney test. Statistics were deemed significant at values *p* < 0.05. Two-way ANOVA analysis was used to test the effects of irisin treatment (first factor) and sex (second factor) on the behavioral parameters. Main effects with a *p* value lower than 0.05 were considered statistically significant.

## Figures and Tables

**Table 1:** Immobility time results in the TST and the FST after short-term and long-term irisin administration.

Type of Treatment	Test	Parameter	Vehicle-Treated Mice (Mean ± SEM)	Irisin-Treated Mice (Mean ± SEM)	Percentage Change
Short-term irisin treatment	TST	Immobility (s)	256.1 ± 12.02	220.5 ± 10.44	−13.90%
	FST	Immobility (s)	201.1 ± 6.323	149.2 ± 11.43	−25.81%
Long-term irisin treatment [28]	TST	Immobility (s)	217.80 ± 11.88	163.80 ± 14	−24.79%
	FST	Immobility (s)	162.33 ± 7.409	133.44 ± 10.39	−17.81%

**Table 2:** Summary of the statistical analysis by two-way ANOVA for the factors: treatment, sex, and treatment x sex.

		Factors		
Behavioral Test	Parameter	Treatment	Sex	Treatment x Sex
TST	Immobility	**F (1.16) = 4.86;** ***p* = 0.042**	F (1.16) = 0.18; *p* = 0.677	F (1.16) = 0.29; *p* = 0.592
FST	Immobility	**F (1.20) = 13.60;** ***p* = 0.002**	F (1.20) = 0.07; *p* = 0.780	F (1.20) = 0.19; *p* = 0.663
EPM	Entries in open arms	**F (1.19) = 4.72;** ***p* = 0.043**	F (1.19) = 0.09; *p* = 0.759	F (1.19) = 0.09; *p* = 0.759
	Time in open arms	**F (1.19) = 4.83;** ***p* = 0.041**	F (1.19) = 0.12; *p* = 0.725	F (1.19) = 0.04; *p* = 0.845
	Entries in closed arms	F (1.19) = 1.70; *p* = 0.207	F (1.19) = 3.47; *p* = 0.078	**F (1.19) = 4.91;** ***p* = 0.039**
	Time in closed arms	F (1.19) = 1.41; *p* = 0.249	F (1.19) = 0.06; *p* = 0.802	F (1.19) = 1.05; *p* = 0.319
	Total stretched-attend posture	**F (1.19) = 4.61;** ***p* = 0.045**	**F (1.19) = 6.51;** ***p* = 0.019**	F (1.19) = 0.12; *p* = 0.729
YM	Total entries	F (1.20) = 0.17; *p* = 0.685	**F (1.20) = 14.76;** ***p* = 0.001**	F (1.20) = 0.13; *p* = 0.724
	Percent alternation	F (1.20) = 2.49; *p* = 0.130	F (1.20) = 0.75; *p* = 0.396	**F (1.20) = 6.19;** ***p* = 0.022**

Significant effects are in bold letters.

**Table 3:** Primer sequences used for quantitative real-time PCR.

Gene Name	Gene Bank Number	Primer Sequence (5′-3′)	Product Size (bp)	Annealing Temperature (°C)
Gapdh	NM_001289726.1	Forward ACACCAGTAGACTCCACGACA Reverse ACGGCAAATTCAACGGCACAG	145	60.48 62.59
Fndc5	NM_027402.4	Forward GTGCTGATCATTGTTGTGGTCC Reverse ATCATATCTTGCTGCGGAGGAG	169	60.10 60.03
Pgc-1α	NM_008904.3	Forward CCCTGCCATTGTTAAGACC Reverse TGCTGCTGTTCCTGTTTTC	161	55.87 56.35

## Data Availability

Not applicable.
